# Bonding Efficiency between Artificial Teeth and Denture Base in CAD/CAM and Conventional Complete Removable Dentures

**DOI:** 10.3390/ma17133138

**Published:** 2024-06-27

**Authors:** Mariya Dimitrova, Angelina Vlahova, Ilian Hristov, Rada Kazakova

**Affiliations:** 1 Department of Prosthetic Dentistry, Faculty of Dental Medicine, Medical University of Plovdiv, 4000 Plovdiv, Bulgaria; angelina.vlahova@mu-plovdiv.bg (A.V.); rada.kazakova@mu-plovdiv.bg (R.K.); 2 CAD/CAM Center of Dental Medicine, Research Institute, Medical University of Plovdiv, 4000 Plovdiv, Bulgaria

**Keywords:** 3D-printing, artificial teeth, CAD/CAM, milling, denture base polymers, complete dentures, adhesion

## Abstract

A common challenge encountered with both traditional and digitally produced dentures involves the extraction of artificial teeth from the denture base. This narrative review seeks to present an updated perspective on the adherence of synthetic teeth for denture base materials, employing diverse methods. Dental technicians often employ chemical approaches and mechanical techniques (including abrasion, laser treatment, and abrasive blasting) to augment the retention of denture teeth. However, the efficacy of these treatments remains uncertain. In certain instances, specific combinations of Denture Base Resin (DBR) materials and artificial teeth exhibit improved performance in conventional heat-cured dentures following these treatments. The primary reasons for failure are attributed to material incompatibility and inadequate copolymerization. As new denture fabrication techniques and materials continue to emerge, further research is imperative to identify optimal tooth-DBR combinations. Notably, 3D-printed tooth–DBR combinations have demonstrated reduced bond strength and less favorable failure patterns, while utilizing milled and traditional combinations appears to be a more prudent choice until advancements in additive manufacturing enhance the reliability of 3D-printing methods.

## 1. Introduction

To meet the social and physiological needs of edentulous patients, complete dentures are frequently employed as an economical solution, replacing the complete set of teeth and associated structures in either the upper or lower jaw [1]. While traditional complete dentures remain the most common approach, modern manufacturing techniques have been implemented in clinical practice to accelerate denture production, simplify production, and lower overall expenses [2,3].

In addition to traditional techniques like heat curing and self-curing, advanced technologies now enable the production of dentures, including both additive and subtractive processes [4,5]. The additive method involves fabricating the denture by building layer upon layer with 3D-printing methods including stereolithography (SLA) or digital light processing (DLP), achieving precise resin layers (ranging from 20 to 150 μm) [6]. Furthermore, additive monolithic manufacturing utilizing material jetting technology has been introduced, enabling the simultaneous printing of different materials with various colors and properties into a single, unified unit. This technique operates by extruding a UV-curable photopolymer liquid in layered form, employing multiple nozzles to handle different materials and colors at once, which are then immediately cured with UV light [7].

Conversely, the subtractive approach employs computer-aided design and computer-aided manufacturing (CAD/CAM), guiding cutting tools via computer numerical control to mill the material. The introduction of additive monolithic manufacturing presents a significant advancement, as it eliminates the need for adhesives by allowing different materials to be printed together in a cohesive unit, thereby enhancing the durability and performance of the denture.

Understanding the types of bonds involved in bonding artificial teeth to denture bases is crucial for optimizing dental prosthetic fabrication techniques. Various bonding techniques are utilized in dentistry, each with distinct characteristics impacting bond strength and durability. In conventional dentures, common bonding techniques include heat curing, self-curing, and microwave curing. Heat polymerization, a form of heat curing, is often favored due to its ability to produce strong bonds [8]. This method involves placing the denture in a heated environment, causing the resin to polymerize and form strong covalent bonds between the denture base and artificial teeth. The heat facilitates optimal resin flow and adhesion, resulting in enhanced mechanical properties and longevity of the denture. In digital workflows, such as CAD/CAM (Computer-Aided Design/Computer-Aided Manufacturing), teeth can be separately fabricated through 3D printing or milling processes before being bonded to the printed denture base. This approach allows for precise customization and alignment of teeth, improving aesthetics and potentially reducing prosthesis failures [9]. The bonding of acrylic teeth to a printed denture base can involve various techniques, including adhesive bonding with resin-based materials or mechanical retention through surface roughening and conditioning. Chemically, these bonding processes rely on interactions such as covalent bonds formed between the resin matrix of the denture base and the bonding agent used to affix the teeth [6]. Covalent bonds involve the sharing of electron pairs between atoms, resulting in strong and stable connections. Surface preparation techniques, such as sandblasting or chemical etching, enhance bonding by increasing surface area and promoting mechanical interlocking or chemical adhesion. Physically, mechanical retention plays a role in bonding strength when teeth are mechanically interlocked with the denture base. This can occur through features like undercuts or grooves in the denture base that physically engage with corresponding features on the artificial teeth [3]. A notable challenge with complete removable dentures is the separation of teeth from the denture base, occurring in 22–30% of conventional removable dentures, particularly around the anterior teeth [10]. The effectiveness of bonding relies on the contact between the denture tooth and the polymerizing denture base material. Improving the adhesive area through surface treatments such as alumina abrasive blasting or macro-retentive elements, such as grooves and inverted cones, enhances the contact region, establishing mechanical interlocking between teeth and denture base [11,12].

With the increased use of dental implants, acrylic resin has become more important in implant restorations [13,14]. Findings indicate that fractures of acrylic teeth are a prevalent complication among patients with prosthetic restorations on implants [15]. With the increased prevalence of osseointegrated implants, the acrylic resin has seen extended use in implant restorations, and highlighting the criticality of the adhesion between dental resin and the PMMA base is essential in restorations such as overdenture implants and combined implant structures [7]. Given their exposure to higher chewing loads, these constructions require high strength to ensure reliability for clinicians.

A variety of commercially available Denture Base Resins (DBRs) and denture teeth are utilized in removable denture manufacturing [16]. However, the specific process used for creating denture teeth can influence their adherence to DBRs. The adhesive strength can be evaluated according to International Standard Organization (ISO) standards, which outline material specifications and testing procedures [17,18]. Despite the introduction of novel manufacturing techniques like CAD/CAM and 3D printing for denture production, there is a current lack of investigations to verify the bonding efficacy of additive and subtractive manufactured denture bases to artificial teeth. Regardless of the method employed, it is crucial to consider the strength of the bond [19,20].

This review aims to provide an up-to-date analysis of the adhesion of denture teeth to resin substrates in traditional and digitally fabricated complete dentures. It also discusses the factors that influence adhesive strength in traditional and modern manufacturing methods.

- Search Strategy and Study Selection

Following the PRISMA guidelines, a comprehensive search of PubMed, Scopus, EMBASE databases, and the grey literature was conducted without imposing any specific time constraints. The search strategy employed a variety of terms related to denture fabrication and adhesion, including ‘artificial teeth’ and ‘bond strength’, ‘complete removable dentures’ and ‘adhesion’, ‘3D printed teeth’ and ‘complete denture’, ‘CAD-CAM teeth’ and ‘complete denture’, ‘milled teeth’ and ‘complete denture’, ‘acrylic teeth’ and ‘complete denture’, and ‘denture teeth’ and ‘denture base’ and ‘adhesion’.

- Inclusion and Exclusion Criteria

The inclusion criteria were focused on retrieving in vitro studies published in English that examined the adhesion, bonding, or detachment of denture teeth and materials. Articles were excluded if they did not provide relevant data on these aspects, or if they focused on removable partial dentures or partial dental prostheses.

Out of the initial pool of 168 articles screened, 138 articles met the following inclusion criteria and were taken into account for the review:

- Written in English;
- Published between 1989 and 2024;
- Focused on the adhesion resistance between artificial teeth and denture base for complete removable dentures, including clinical and in vitro studies;
- Provided information on various manufacturing methods, properties, and clinical assessments involving traditional, 3D–printed, and milled complete removable dentures.

- Manual Search

**Figure 1:**
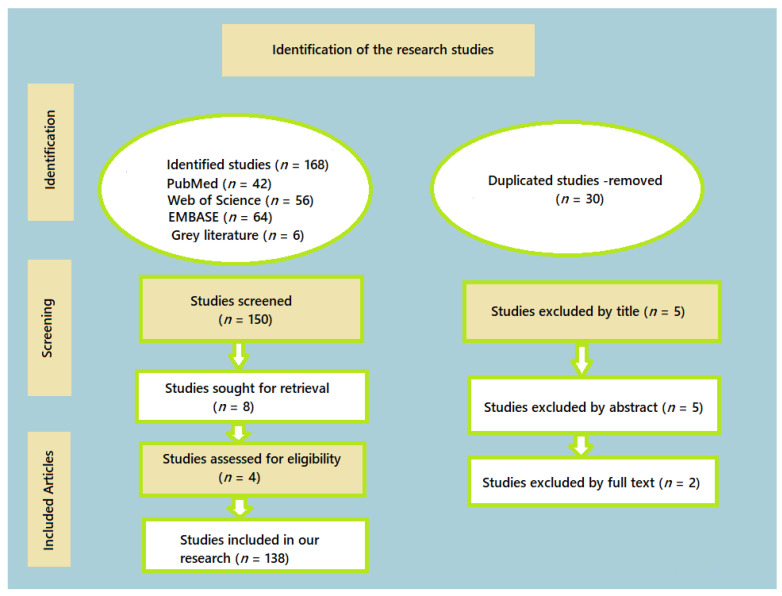
The configuration of the study undertaken.

To ensure the comprehensiveness of our review, we also conducted a manual search of all included articles and relevant reviews. This additional step aimed to identify any pertinent studies that may have been missed in the initial database searches (Figure 1).

- Selection Process

Throughout the selection process, we meticulously documented the number of articles selected and refined at every stage. The period from 1 January 1993 to 31 April 2024, was chosen because of the heightened accessibility of clinical and in vitro studies during this time frame. This thorough methodology ensured that only the pertinent and superior literature was included in the review. Table 1 lists the articles that were selected, highlighting those that were most pertinent to the subject matter.

## 2. Mechanism of Adhesion of Artificial Prefabricated Teeth to Traditional PMMA Denture Base

From a standpoint in clinical practice, the dislodgment of teeth from removable complete or partial dentures, particularly in the anterior region, can lead to patient discomfort and often requires immediate dental attention. The causes of tooth detachment may arise from errors in the denture manufacturing steps, issues related to the types of materials used (both teeth and denture base resin), or excessive forces applied during chewing [21]. The long-term reliability of these prosthetic devices is crucial for achieving greater patient satisfaction [22].

Reports indicate that a substantial number of denture repairs, exceeding 60% of annually produced dentures, are linked to issues of tooth detachment [19]. Similar findings are reiterated in other studies, emphasizing that one of the most prevalent types of repairs involves the debonding of artificial teeth in removable dentures [23]. While excessive pressure on the occlusal surface during chewing may contribute to tooth detachment, the literature suggests the presence of other contributing factors.

A significant explanation for the breakdown of the adhesion between denture teeth and the base is associated with surface contamination on the teeth [24], impeding the establishment of a strong bond. Contaminants typically include traces of wax absorbed during polymerization or residual material left at the base of the artificial teeth during the denture’s investment. However, Spartley’s research contradicts this perspective, indicating that residual material on teeth does not significantly affect adhesion strength if wax elimination is performed at temperatures of at least 90 °C [8]. Debonding may also result from variations in surface properties where the tooth meets the base. This incompatibility is believed to arise from either surface contamination or structural disparities between the two components due to distinct fabrication methods [22,23,24,25]. Research indicates that tooth detachment contributes to 22–30% of denture repairs, especially in the front section, although there is no differentiation made between fracture and debonding [26].

The repair process may involve creating an inverted cone within the denture tooth, a technique commonly employed by many dental technicians before introducing heat-cured acrylic, to address these challenges more effectively [27,28]. Another contributing factor may involve the routine use of disinfectants or cleaning chemicals, which have the potential to alter the mechanical and physical characteristics of both DBR and the teeth [29,30,31]. This alteration can lead to a weakening of the bond between them, resulting in detachment [32].

Concerning material-related aspects impacting the connection between artificial denture teeth and Denture Base Resin (DBR) materials, these factors include differences in the composition and polymerization technique of the denture base, the type of acrylic teeth employed, and the preparation of their interfacial surfaces [33]. The primary process governing the adhesion involves the expansion of the polymer induced through the dispersion of a suitable solvent. The rate of diffusion is dependent on factors such as time, temperature, solvent, polymer structure, and the polymer’s glass transition temperature [34].

Since denture teeth are pre-polymerized, achieving chemical co-polymerization to establish interconnected polymer chains with any denture base material is challenging due to the very low concentration of free radicals [22]. Additionally, the adhesion of denture base resin to denture teeth is attributed to the involvement of unreacted methyl-methacrylate groups. However, this cannot be verified in cold-cured materials because residual double bonds remain unreacted at room temperature [35]. Although PMMA conventional acrylic achieves a high level of double-bond conversion and generates a significant number of reactive free radicals, the low concentration of free radicals in pre-polymerized resin teeth does not guarantee adhesion to PMMA denture bases [36]. To address this challenge, several approaches have been explored to enhance the bond strength at the interface. These approaches involve the creation of micro- or macro-mechanical interlocking or the initiation of some form of a chemical interaction occurring between various polymer types [37].

## 3. Mechanism of Adhesion of 3D-Printed and Milled Artificial Teeth to Denture Base

In the conventional production of removable dentures, the adhesion between denture bases and teeth is accomplished via PMMA polymerization, which happens upon contact with the artificial teeth, resulting in an intertwined polymer network. Nonetheless, the digital denture process usually entails digitally designing and separately manufacturing bases and artificial teeth, subsequently bonding them together using a bonding agent, surface conditioning, or the usage of auto-polymerizing PMMA resin [38].

**Figure 2:**
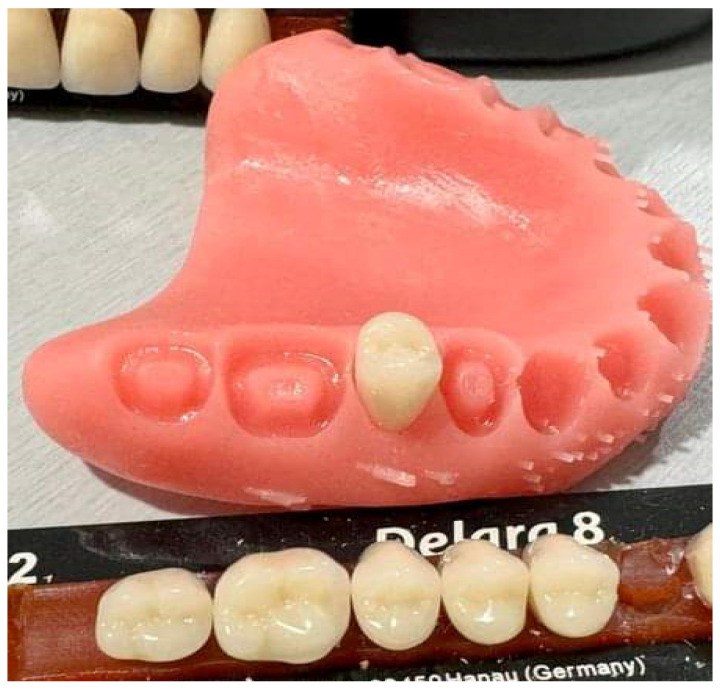
Artificial prefabricated tooth—second premolar (Delara, Heraeus Kulzer, Hanau, Germany) placed in the socket of the 3D-printed upper denture base (NextDent Denture 3D+, NextDent, 3D Systems, Soesterberg, The Netherlands). Source of the figure: clinical case from the author; no copyright concerns.

Pre-made or milled teeth are bonded to the denture base either through adhesive application or by bonding with resin cured through cold and heat methods. With 3D-printed dentures, the base is initially printed, followed by the separate printing of teeth in a subsequent stage [39]. The artificial teeth can be attached to the denture base utilizing a designated adhesive agent or resin that has not undergone polymerization, which is then solidified using light. The teeth can be bonded individually or fused and bonded as one unit (Figure 2). Alternatively, artificial denture teeth can be adhered to a printed denture base using specialized bonding agents and sealants [40].

**Figure 3:**
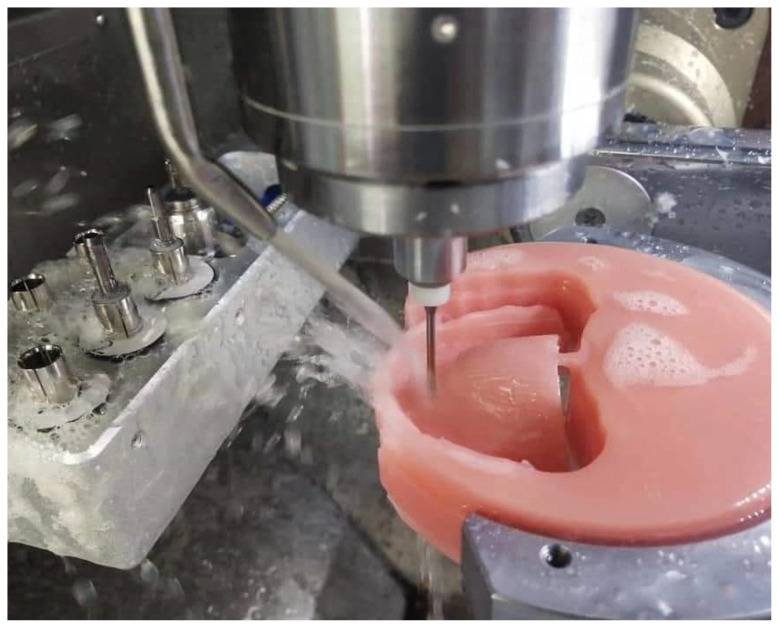
Fabrication of a complete removable denture using a PMMA disc through a milling process (Ivotion Denture System, Ivoclar Vivadent, Schaan, Liechtenstein). Source of the figure: clinical case from the author; no copyright concerns.

Lately, there have been developments in monolithic digital denture solutions. The additive technique revolutionizes manufacturing by facilitating the simultaneous printing of an array of materials, each possessing unique colors and properties, that are seamlessly fused into a single, unified entity [7]. Manufacturers now provide teeth and denture base materials, both made of high-quality PMMA, on two-toned discs (pink and white) (Figure 3). These materials are intended to undergo milling together in a unified milling operation [41]. Throughout the industrial manufacturing process, both materials are polymerized concurrently, creating a direct chemical adhesion and removing the necessity for additional bonding materials and processes [42,43].

**Figure 4:**
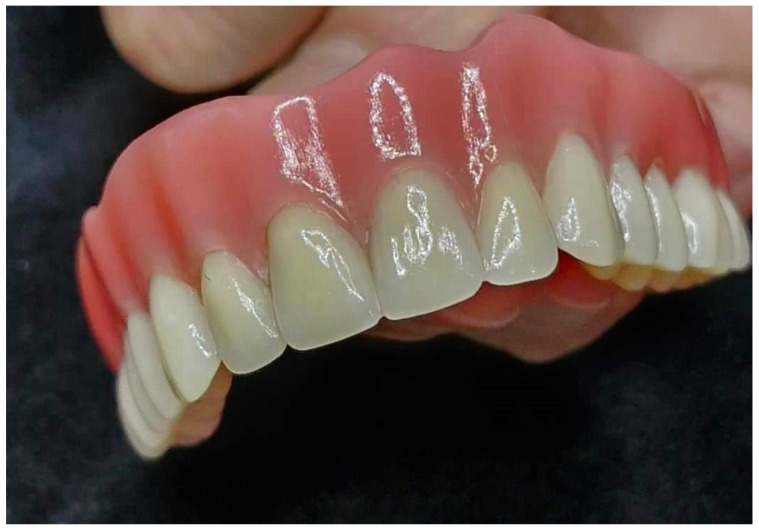
A 3D-printed denture with a separately manufactured base and artificial teeth (NextDent Denture 3D+, NextDent, 3D Systems, Soesterberg, The Netherlands). Source of the figure: clinical case from the author; no copyright concerns.

The recent literature indicates that the connection between 3D-printed denture bases and separately printed teeth is less robust compared to traditional methods (Figure 4) [44,45,46]. One study demonstrated that the printed group exhibited both cohesive and adhesive breakdowns, while the traditionally processed group only showed cohesive failures, implying a stronger bond in the conventionally processed denture group [47]. Despite limited investigation in this domain, it seems that printed dentures generally display diminished bond strength. Further research is necessary to evaluate whether this has clinical relevance, and a comparison of different adhesive techniques should be undertaken, as several adhesive procedures have been suggested [35,48].

Presently, there are limited in vitro investigations examining the adhesion strength of denture teeth across heat-processed and contemporary-processed denture bases. Choi et al. [49] conducted a study comparing four distinct varieties of commercial denture teeth (PMMA, cross-linked PMMA, PMMA incorporating nanofillers, and 3D printed) with three types of DBRs (heat-cured, milled, and 3D printed). Their results revealed that heat-polymerized denture base resins still offer the greatest bond strength and fracture toughness, notwithstanding the growing popularity of CAD-milled and 3D-printed materials [50].

Prpić et al. [51] conducted a study comparing the shear bond strengths between various denture base resins and different types of prefabricated teeth, including acrylic, nanohybrid composite, and cross-linked teeth, as well as CAD/CAM-produced denture teeth. Their findings revealed that cold-cured resin exhibited the lowest values among the different polymerization methods [52].

Remarkably, there was no significant difference in shear bond strength values between CAD/CAM (milled) denture base resins and heat-cured resins. The bond strengths were comparable when milled and heat-cured denture base resins were bonded to various types of prefabricated teeth [53]. This suggests that the primary factor influencing bond strength was the polymerization process of the denture base resin. Given the variability in bonding strength between removable denture bases and denture teeth based on material combinations, the authors recommended avoiding the use of cold-cured resin for attaching prefabricated teeth to a denture base [54,55].

## 4. Approaches to Enhance the Adhesion Resistance between Denture Base and Artificial Teeth

Researchers have explored various methods to enhance the attachment between denture base and teeth. Most of these approaches require preparation of the surface in the bonding region of the artificial tooth [56,57,58]. The strength of the connection is affected by both the type of artificial teeth and the composition of the denture bases [59]. In simple terms, the chemical makeup of pre-polymerized artificial denture teeth influences the surface treatment process. Additionally, the composition of the denture base material and the polymerization techniques utilized also affect the bonding capability of pre-made artificial teeth.

Typically, approaches to modifying the surface of a prefabricated artificial tooth are grounded in principles of micro-mechanical retention, chemical co-polymerization, and managing polymerization shrinkage in polymers [39,60].

Chemical treatments function by utilizing polymerizable monomers to soften the surface of the acrylic tooth, enabling them to permeate the acrylic material. Monomers from the polymerized resin of the base material then infiltrate the acrylic resin of the denture teeth, causing surface expansion [61]. The thickness of this layer, resulting from the interaction of monomers with polymer particles and the resin matrix, appears crucial for the strength of adhesion between the PMMA of the teeth and the base resin. Upon polymerization, these monomers form a network of polymer chains that interconnect the two polymers [62]. Various chemical modifications have been explored to improve the bond between teeth and denture base materials (Table 2).

Numerous researchers have explored different treatments for acrylic teeth, including the application of liquid methyl methacrylate (MMA) [62,63,64,65] or a mixture of MMA and methylene chloride [66,67,68]. Spratley et al. [8] found that the placement of monomer on the cervical region of the teeth did not seem to impact bond strength. Similarly, Barpal et al. [69] observed that treatment of the surface of acrylic teeth with MMA monomer either decreased bond strength or had no discernible effect on the adhesion resistance of the thermosetting PMMA resin to the denture base when applied for 30 s prior to applying the resin layers.

**Table 2:** Investigations assessing the adhesion between artificial teeth and denture base substances.

Type of Artificial Teeth	Type of Denture Base Material	Type of Chemical Treatment	Type of Mechanical Treatment	References
PMMA teeth	Heat-polymerized resin Auto-polymerized resin	MMA, 180 s	Using 120-grit sandpaper for grinding, create two grooves and a retention hole with a diameter of 1.5 mm, F = 10 MPa	Vallittu et al., 1997 [22]
PMMA teeth Composite teeth Nanocomposite teeth	Heat-cured resin	MMA	N/A	Gharebagh et al., 2019 [56]
3D-printed teeth, Prefabricated acrylic teeth	3D-printed denture resin, Heat-cured resin	MMA, 3D-printed resin, Auto-polymerized acrylic resin	400–1200-grit SiC paper, F = 10 MPa	Cleto et al., 2022 [63]
PMMA teeth, Composite teeth	Heat-cured resin, CAD/CAM-milled	DCM, PMMA-based bonding agent	Roughening with bur 250 m Al_2_O_3_, 15 s, 4.8 bars, 10 mm, F = 10 MPa	Helal et al., 2022 [30]
PMMA teeth, 3D-printed teeth	Heat-cured resin, Milled PMMA resin 3D-printed resin	Self-curing Bonding agent, Uncured 3D-printing resin	N/A	Choi et al., 2020 [49]
PMMA teeth	Heat-cured resin	MMA DCM	250 m Al_2_O_3_, 4.8 bars, 5 s, 5 mm 5 mm, F = 10 MPa	Viegas et al., 2021 [53]
3D-printed teeth, Prefabricated composite teeth, Milled teeth	Heat-cured resin Milled PMMA resin 3D-printed resin	3D tooth conditioning agent, 4 min, 40 C + light-cured bonding agent	N/A	Mohamed et al., 2022 [43]
PMMA teeth Artificial teeth Milled teeth	Heat-cured resin Cold-cured resin PMMA resin produced by milling	PMMA-based bonding	N/A	Prpić et al., 2020 [51]
Acrylic teeth	Heat-polymerized resin	MMA	50 mm Al_2_O_3_, 20 s Diatoric cavity 1.5 mm, F = 10 MPa	Barpal et al., 1998 [69]
Acrylic teeth	Auto-polymerized resin	Methyl methacrylate (MMA), 3 min Composite bonding agent 37% phosphoric acid etchant + Methyl methacrylate MMA + composite bonding agent	F = 10 MPa, Grinding at low speed (0.5 mm/s)	Yanikoglu et al., 2002 [25]

Morrow et al. [70] indicated that applying a monomer–polymer mixture to the unmodified cervical region of denture teeth reduced the bond strength, which aligns with findings by Dimitrova et al. [71], who observed decreased bonding when monomer was applied to the tooth surface with self-curing acrylic resin. Palitsch et al. [72] found that using MMA in combination with light-cured denture base materials resulted in inadequate adhesion performance, consistent with the existing literature. They attributed these outcomes to factors such as the limited penetration of denture base resin (DBR) into MMA-treated tooth surfaces, the viscous nature of the denture base material hindering micromechanical interlocking, or the insufficient copolymerization between MMA and the bifunctional monomers in light-cured DBR materials [73,74]. These variations in the results can be attributed to differences in testing methods and the materials used for denture base and teeth.

Apart from MMA, various chemical substances have been investigated. Sorensen and Fjeldstad [75] reported an improved bond resistance when acrylic teeth were treated with suitable solutions like ethyl acetone or monomer. Takahashi et al. [76] discussed the beneficial effects of dichloromethane (CH_2_Cl_2_), an organic solvent capable of breaking down PMMA polymer structure, on enhancing the retention of both conventional and cross-linked acrylic teeth. In a related study, dichloromethane significantly increased the tooth bond strength, tripling the initial value observed in untreated teeth. This solvent expanded the external surface volume of acrylic resin in the teeth, facilitating the infiltration of polymerizable acrylic resin monomers from the base into artificial teeth and forming a comprehensive interlaced polymer network [77,78]. The increased mechanical retention resulting from the micro-roughness observed on tooth surfaces treated with dichloromethane may contribute to the higher bond strength [79]. Suzuki et al. [80] observed a significant improvement in bonding when administering 4-META adhesive agents on extensively cross-linked teeth before packing the resin dough. Fletcher-Stark et al. [81] studied a notable increase in bond strength in IPN denture teeth when an adhesive agent (Eclipse, Ottawa, ON, Canada; Dentsply, Charlotte, NC, USA) was used in conjunction with a light-polymerized UDMA resin, but this enhancement was not observed when paired with a heat-polymerized PMMA resin.

Another adhesive agent utilized for attaching the denture base materials to acrylic teeth is methylacetate, commercially known as Eclipse Bonding Agent (Eclipse; Dentsply) [82]. In research by Akin et al. [83], its application did not yield an optimal bond strength when paired with a conventional denture base. Nonetheless, in a subsequent investigation conducted by the same researchers [84], significant improvements in bond strength were observed when it was paired with light-cured denture base materials. The same bonding agent was tested alongside a dichloromethane-based bonding agent for its effectiveness in bonding to a light-cured DBR. It demonstrated effectiveness only when utilized alongside a surface that had been mechanically roughened [85].

Nishigawa et al. [86] used an adhesive bonding substance composed of 85% MMA and 15% low-molecular-weight polyethylmethacrylate (PEMA). They found that this adhesive significantly increased the shear bond strength between DBR and artificial denture teeth, even without sandblasting, and maintained the highest bond strength even after 100 days of water immersion. Specifically, interfaces that were sandblasted and treated with this additional adhesive agent retained their strength notably well after prolonged water exposure. Perea et al. [87] conducted experiments using four different monomer systems to evaluate the shear bond strength between PMMA denture base resin (DBR) and acrylic resin denture teeth. These systems included flowable composite resin, methyl methacrylate (MMA), stick resin, and composite primer. All materials, except MMA, contained a photopolymerizable initiator system. It was observed that a strong bond could be achieved when these monomer systems were given sufficient time to effectively dissolve the polymer network of acrylic teeth [88,89]. Furthermore, there have been suggestions to improve the overall contact surface area between the two polymer materials by adjusting macro-mechanical retention and roughness. Sandblasting, a conventional technique used before cementing base metal and zirconia restorations, is a common method to achieve this [90]. It appears that sandblasting improves the bonding strength of both denture teeth made of acrylic and ceramic. Other approaches involve altering the adhesive region through mechanical abrasion or by creating macro-retentive patterns. Research has shown that vertical grooves reduce stress concentration at the interface between the tooth and base material [91,92]. Additionally, the inclusion of two aligned grooves and one retention aperture has been found to enhance the tensile strength of denture teeth in the base material. An investigation conducted by Akin et al. [84] did not utilize grooves but ground the bonding surface with a tungsten carbide bur for generating regions with macro-retentive features. They also explored alternative mechanical treatments, such as Er: YAG laser and airborne-particle abrasion using 120 µm Al_2_O_3_ particles at a pressure of 2 bar for 10 s and found that all these mechanical pretreatment methods effectively improve the adhesion strength. Chung et al. [93] integrated sandblasting using 250 µm Al_2_O_3_ particles with grinding and observed that this combination notably enhances the bond strength due to the increased surface area and mechanical interlocking [94].

On the other hand, Cardash et al. [95] observed varying effects on bond effectiveness in artificial teeth with a mechanically altered cervical portion, with both increases and decreases noted. Cunningham et al. also mentioned that adding grooves and grinding the tooth surface can be effective even in the absence of a thorough dewaxing [96].

## 5. Impact of Various Denture Base Materials on Bond Efficiency

Denture base materials are typically classified based on their method of polymerization [97]. Several types of denture base materials have been developed, including heat-cured, visible-light-cured, microwave-cured, cold-cured, and pour-type denture base materials. Poly (methyl methacrylate) (PMMA) is the most commonly used material for Denture Base Resin (DBR) due to its widespread acceptance, despite its relatively low mechanical strength [85]. To improve the mechanical properties of PMMA denture base resin (DBR) and reduce the risk of fractures, various additives have been explored. These include rubber, glass, polyethylene, and polypropylene fibers, as well as alumina, titanium, zirconia, silver, silica-based, and hydroxyapatite fillers [98].

One drawback of PMMA is its potential to cause allergies in patients sensitive to the methyl methacrylate monomer [99]. As an alternative to PMMA, light-polymerized resins were introduced, which consist of dimethacrylates such as Bis-GMA, TEGDMA, and UDMA [100]. However, these light-polymerized resins have shown a lower bond strength with denture teeth, often requiring the use of bonding agents [101]. Composite materials offer advantages such as easier processing and a reduced risk of allergic reactions [102]. Depending on the specific material used, particular conditioning solutions are recommended for chemically preparing resin denture teeth. The primary function of a conditioning liquid is believed to be its ability to penetrate the bonding surface and solubilize and/or swell it, thereby facilitating a chemical bond with the denture base material [79,83].

Artificial teeth generally form stronger bonds with heat-cured acrylic resins compared to self-cured acrylic resins. This is attributed to the more thorough polymerization process in heat-cured acrylic resins, as noted by some researchers [89]. Others suggest that the higher polymerization temperature in heat-cured acrylic resins promotes monomer diffusion into the teeth, resulting in an enhanced adhesion performance [103].

The various types of artificial teeth and the different materials used in denture bases influence the strength of their bond. Recently introduced highly cross-linked artificial teeth offer significantly improved properties such as an enhanced fracture resistance, abrasion resistance, and color stability [104]. However, traditional acrylic teeth typically establish a stronger bond with the resin base of the denture compared to highly cross-linked acrylic teeth. To ensure the copolymerization process during the manufacture of acrylic teeth, manufacturers incorporate a less cross-linked polymer in the cervical part. According to a study [105], conventional acrylic teeth have more non-crosslinked polymer chains available for bonding to the denture base.

Clancy et al. [89] evaluated the adhesion strength of light-cured and heat-cured resins with both conventional acrylic teeth and abrasion-resistant teeth. They found that the most effective combination was heat-cured resin with conventional acrylic teeth, exhibiting a higher bond strength compared to abrasion-resistant teeth with IPN (interpenetrating polymer network) properties. In contrast, the bond strength with light-cured resin was consistently lower for both types of teeth [106]. In the study conducted by Saponaro et al. [107], a pour-type Denture Base Resin (DBR) was bonded to both traditional and cross-linked denture teeth and evaluated before and after undergoing thermal cycling. The researchers found no significant differences between the groups, either before or after thermal cycling. They highlighted the effectiveness of dichloromethane in improving adhesion strength even after thermal cycling. Dichloromethane works by causing swelling in the resin of denture teeth, which allows deeper penetration of monomers and strengthens their network. Additionally, it induces micro-roughening on the surface, thereby enhancing micromechanical bonding [108]. Evaluating the adhesion resistance of each possible combination is a substantial undertaking, given the extensive array of teeth and denture base materials available in the market. Instead, a more effective approach might involve assessing the bonding characteristics of both “matched” and “mismatched” combinations of teeth and denture base materials [109]. There is limited literature suggesting that a stronger bond may be attained when both the Denture Base Resin (DBR) and denture teeth are manufactured by the same company [110,111,112]. In a study conducted by Tanoue et al. [113], NHC teeth with various DBR materials were tested, which showed notably lower adhesion performance when paired with denture bases from different manufacturers, despite their chemical similarity.

## 6. Discussion

This narrative review has summarized the findings from in vitro studies investigating the bonding effectiveness between traditional and modern combinations of artificial teeth and denture base materials. While there is an abundant literature on studies involving traditional materials and methods, research on CAD/CAM and 3D-printed teeth remains scarce. However, challenges in synthesizing and extrapolating findings arise from variations in experimental design, specimen preparation, material combinations, sample sizes, and other factors.

In the conventional process of fabricating removable full dentures, denture base resins derived from methyl methacrylate monomers can be polymerized into PMMA using methods such as heat, chemical agents, visible light, and microwave energy [114]. Despite their relative speed, these methods often leave residual free monomers due to incomplete polymerization. Additionally, heat-polymerized dentures may exhibit porosities caused by factors like inadequate mixing, excessive heating, evaporation of unreacted monomer, or insufficient pressure during polymerization [115]. In contrast, milled dentures are crafted from pre-polymerized PMMA blocks known for their minimal shrinkage, high density, and low porosity. These materials lack residual monomers because of their more thorough transformation process and effective polymerization. It has been noted that free monomers present during manufacturing can affect the adhesive strength between denture teeth and Denture Base Resins (DBRs) [116]. Consequently, traditional processing methods may provide better adhesion compared to bonds formed with pre-polymerized modern materials [117]. Choi et al. [49] found that following the manufacturer’s instructions for conventional and CAD/CAM complete denture fabrication results in a 2.5-fold increase in free monomer concentration at the interface, initiating the bonding process in heat-polymerized dentures compared to pre-polymerized CAD/CAM materials. This higher concentration of free monomers could explain the weaker bond strength observed in CAD/CAM specimens in their study. Specific research indicates that the bond strength between artificial teeth and heat-cured resins is greater than that of self-cured acrylic resins [118,119,120,121]. In a study by Takahashi et al. [76], heat-cured and microwave-processed Denture Base Resins (DBRs) were compared with a poured PMMA DBR resin. The heat-cured DBR exhibited the best results, highlighting a significant difference. Both heat-cured and microwave-processed DBRs exhibited a superior bond strength compared to pour-type resin [122,123,124,125]. Similar findings were reported by Damade et al. [126] in their tensile tests, where they assessed the bonding of cross-linked acrylic teeth using heat and microwave polymerization methods. The materials utilized for artificial teeth in both removable complete and partial dentures primarily comprise heat-activated poly (methyl methacrylate) (PMMA) grains integrated within a cross-linked PMMA matrix [127]. Additionally, highly cross-linked polyacrylic resins incorporate uniformly dispersed inorganic microfillers that polymerize within the matrix, forming materials such as interpenetrating polymer networks (IPN) or nanohybrid composite resins. These include blends of UDMA matrix with inorganic SiO_2_ fillers and clusters of PMMA [128].

The manufacturing method that builds dentures by adding material layer by layer can introduce flaws and pores due to inadequate packing between the layers. These imperfections have the potential to impact the mechanical performance of the bonded interface [129,130]. It has been proposed that the orientation of applied loads, particularly concerning the printing configuration, could heighten the risk of artificial teeth detachment, frequently leading to adhesive-type fractures at the interface [131]. Cleto et al. [63] have proposed that using a methyl-methacrylate monomer may be more effective compared to other bonding agents like auto-polymerizing or 3D-printing resin. However, the monolithic additive manufacturing method successfully addresses these challenges linked with conventional layer-by-layer fabrication techniques, particularly the problem of bonding teeth to the denture base [132,133]. By employing a unified printing process, this technique eradicates the necessity for adhesives entirely. This seamless integration is accomplished by meticulously layering materials, resulting in the teeth and base melding into a unified structure with each successive layer [134,135,136]. Consequently, the prevalent risks of separation or detachment between teeth and base, typically encountered in traditional denture construction, are essentially eradicated [137,138].

Given the conflicting findings observed in the limited studies, there is a clear necessity for further research to determine if there is a consistent trend in the prevalence of modern techniques over traditional ones. Furthermore, the inconsistencies in methodologies used to assess the actual bond strength between denture teeth and denture bases, combined with irregular adherence to ISO guidelines, hinder the ability to systematically analyze the findings across the studies included.

## 7. Conclusions

In summary, ongoing debates persist regarding the most effective combination, as different studies yield conflicting results. Some studies indicate that composite teeth bond to PMMA base materials more effectively than acrylic teeth, while others propose the opposite. Future research should explore new materials and processes to address the problem of artificial teeth detachment from denture bases. Moreover, the increasing popularity of 3D-printing removable dentures, thanks to their ease of production, the capacity to create complex dentures, and cost-efficiency, underscores the necessity to optimize bonding protocols for modern 3D-printed teeth to ensure long-lasting bonds.

## Figures and Tables

**Table 1:** The most relevant articles incorporated in the study.

Author’s Name	Year of Publication	Type of Study	Manufacturing Technique
Matos et al. [21]	2018	In vitro study	Microwave-cured acrylic resin denture base
Vallittu, P.K.; Ruyter, I.E. [22]	1997	In vitro study	Acrylic resin polymer teeth and denture base polymers
Kiteska et al. [23]	2018	In vitro study	Dental composites
Burtscher, P. [24]	1993	In vitro study	Cured composite materials
Yanikoglu et al. [25]	2002	In vitro study	Autopolymerizing denture resin and light cured composite resin
Adeyemi et al. [26]	2007	In vitro study	Acrylic tooth-denture base bond
Baghani. et al. [27]	2018	Literature review	Various factors affecting bonding strength
Boonpitak et al. [28]	2022	In vitro study	3D-printed artificial acrylic teeth and denture base resins
Al-Somaiday et al. [29]	2022	In vitro study	Surface modifications of acrylic teeth and polycarbonate denture base material
Helal et al. [30]	2022	In vitro study	Artificial teeth bonded to denture base resins

## Data Availability

No new data were created or analyzed in this study.

## Abbreviations

CAD/CAM	Computer-aided design and computer-aided manufacturing
CCD	Conventional complete dentures
DCM	Dichloromethane
DBR	Denture base resin
DCMA	Glacial acetic acid
DLP	Digital light processing
GA	Glacial acetic acid
IPN	Interconnected penetrating network
ISO	International Standard Organization
MA	Methacrylic acid
MMM	Monomethyl methacrylate monomer
MMRP	Microfillers polymerized into the matrix
MMA	Methyl-methacrylate
PEMA	Polyethylmethacrylate
PMMA	Polymethyl-methacrylate
SBS	Shear bond strength test
SLA	Stereolithography
TCM	Trichloromethane
UDMA	Urethane-dimethacrylate
